# Intraoperative Localization of STN During DBS Surgery Using a Data-Driven Model

**DOI:** 10.1109/JTEHM.2020.2969152

**Published:** 2020-01-30

**Authors:** Mahsa Khosravi, S. Farokh Atashzar, Greydon Gilmore, Mandar S. Jog, Rajni V. Patel

**Affiliations:** 1 Department of Electrical and Computer EngineeringUniversity of Western Ontario 6221 LondonONN6A 3K7Canada; 2 Canadian Surgical Technologies and Advanced Robotics (CSTAR)Lawson Health Research Institute 151158 LondonONN6A 4V2Canada; 3 Department of Electrical and Computer EngineeringTandon School of EngineeringNew York University 5894 New YorkNY10003USA; 4 Department of Mechanical and Aerospace EngineeringTandon School of EngineeringNew York University 5894 New YorkNY10003USA; 5 NYU WIRELESS, Tandon School of EngineeringNew York University 5894 New YorkNY10003USA; 6 School of Biomedical EngineeringUniversity of Western Ontario 6221 LondonONN6A 3K7Canada; 7 Department of Clinical NeurosciencesUniversity of Western Ontario 6221 LondonONN6A 3K7Canada; 8 London Health Sciences Centre 10033 LondonONN6A 5W9Canada

**Keywords:** Deep brain stimulation, deep neural network, intraoperative localization of STN, Parkinson’s disease

## Abstract

A new approach is presented for localizing the Subthalamic Nucleus (STN) during Deep Brain Stimulation (DBS) surgery based on microelectrode recordings (MERs). DBS is an accepted treatment for individuals living with Parkinson’s Disease (PD). This surgery involves implantation of a permanent electrode inside the STN to deliver electrical current. Since the STN is a very small region inside the brain, accurate placement of an electrode is a challenging task for the surgical team. Prior to placement of the permanent electrode, microelectrode recordings of brain activity are used intraoperatively to localize the STN. The placement of the electrode and the success of the therapy depend on this location. In this paper, an objective approach is implemented to help the surgical team in localizing the STN. This is achieved by processing the MER signals and extracting features during the surgery to be used in a Machine Learning (ML) algorithm for defining the neurophysiological borders of the STN. For this purpose, a new classification approach is proposed with the goal of detecting both the dorsal and the ventral borders of the STN during the surgical procedure. Results collected from 100 PD patients in this study, show that by calculating and extracting wavelet transformation features from MER signals and using a data-driven computational deep neural network model, it is possible to detect the borders of the STN with an accuracy of 92%. The proposed method can be implemented in real-time during the surgery to model the neurophysiological nonlinearity along the path of the electrode trajectory during insertion.

## Graphical abstract

We present new techniques to assist neurosurgeons during deep brain stimulation (DBS) surgery by providing accurate, real-time localization of the subthalamic nucleus (STN). A combination of discrete wavelet transformation features and a deep neural network algorithm are suggested as a highly accurate approach to localize the STN during DBS surgery with an accuracy of 92%.

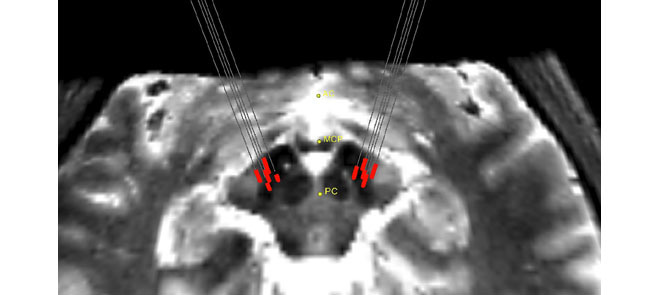

## I. Introduction

Parkinson’s disease (PD) is a progressive neurological disease that affects 1% of people over 60 years of age [1], [2]. Motor features of PD result from the death of dopamine neurons in substantia nigra pars compacta of the Basal Ganglia (BG). Oral pharmacotherapy and surgical intervention are both accepted as treatments. Deep Brain Stimulation (DBS) surgery is an effective therapy used for neuropsychiatric disorders especially in those that have advanced PD [3], [4]. During DBS surgery, a permanent electrode is implanted inside the brain to deliver high-frequency electrical pulses to the subthalamic nucleus (STN) [5]. The outcome of DBS surgery is highly dependent on the accurate placement of the electrode inside the STN. Since the STN is a very small (4–7mm) and deep anatomical region, appropriate and accurate implantation of the electrode is a difficult, challenging and time-consuming task that requires a high level of proficiency and expertise. Due to the sensitivity and importance of implantation, significant intraoperative time is spent on localizing the borders of the STN. In fact, sub-optimal positioning of DBS electrodes accounts for 40% of cases of inadequate efficacy of stimulation post operation [6]. In current practice, preoperative Magnetic Resonance Imaging (MRI) is used to locate the STN according to a visual atlas [7]. However, the exact location of the STN cannot be determined from MRI images [8]. Thus, intraoperative Micro Electrode Recordings (MERs) are also used to localize the STN using electrophysiological properties of the brain tissue surrounding the STN and within the STN itself. In a typical DBS surgery, up to five microelectrodes are inserted through a burr hole in the skull. The microelectrodes record the electrophysiological activity along a track as they are sequentially advanced into the brain by the neurosurgeon [9]. Since each part in the brain has its own characteristic neural activity, that of the STN (such as spike firing counts and patterns) can be recognized over the background noise level. As a result, based on monitoring of this electrophysiological activity, the neurosurgeon decides when the microelectrode has entered the STN [7], [10].

The topic of STN localization accuracy using electrophysiology has been studied in the literature, and several techniques have been implemented (e.g., [11]–[13]). A complete review survey has been published recently on all the studies conducted so far that used different feature extraction techniques and machine learning algorithms for localizing the STN nucleus [14]. In [14], Wan *et al.*, have reported a complete summary of the state-of-the-art algorithms that have achieved good accuracies. It is mentioned that most of the existing results are not robust enough for clinical implementation and further research is needed on the topic of detecting the STN nucleus. Some major issues of localizing the STN during DBS surgery in the literature have been elaborated in [14]. One of the most important issues is with real-time implementation of localizing the STN. postoperative processing steps are needed (such as some critical normalization) to prepare the feature space to be used for classification. These methods cannot be used intraoperatively [14]. In [11] multiple computational features have been suggested to identify the dorsal border of the STN using an unsupervised machine learning algorithm. This work was completed in 2015 [13] with the development of a new feature selection and normalization method that is based on the previously-suggested features. In [13], ten features were suggested as the best features to use in the classification problem. In addition, four classifiers were evaluated in [13]. Among the suggested algorithms, the Logistic Regression (LR) algorithm was reported as the most accurate scheme. In addition, in [12], four features were selected from [11], and a Support Vector Machine (SVM) technique was used as the classifier. In addition, Moran *et al.* [15], have shown the feasibility of estimating entry and exit points of the STN based on normalized Root Mean Square (RMS) values.

Although high performance has been reported in some of the aforementioned articles, the existing high-performance techniques cannot be implemented in the operating room and *during* surgery. The reason is that the extracted features used in conventional techniques require post-operative processing steps (such as spike sorting and a specific normalization algorithm that requires information from the whole insertion trajectory) [11], [13]. However, the existing approaches can be used as post-operative validation techniques (which can help to evaluate the quality of the conducted operation after completion of the surgery). However, they do not allow for STN localization in an intraoperative manner. As a result, they cannot be used as a tool to provide feedback to the surgical team intraoperatively for enhancing the quality of concurrent surgery. In a preliminary study, based on data from five patients, Cardona *et al.* [16], evaluated the idea of using features without normalization and creating an online platform for STN localization. They suggested that normalization of features not be used since normalization results in loss of high-frequency components of signals which can be informative in detecting the STN. However, they have reported that due to the challenging nature of locating the STN during DBS surgery, the accuracy of online techniques is significantly lower than that of offline techniques. It is mentioned that high accuracies are not guaranteed and further analysis should be conducted on more data to enhance accuracy of online systems [16]. In a recent paper [17], Valsky *et al.*, have used Normalized Root Mean Square (NRMS) and Power Spectral Density (PSD) to detect the ventral border of STN (the ending border) using a Support Vector Machine and a Hidden Markov Model. In that paper, high accuracy is reported for the exit boundary of STN (0.04 ± 0.18 mm) on 131 microelelectrode trajectory recordings. The proposed method in [17] requires Normalized RMS which indicates that it is not feasible to implement intraoperatively for both entry and exit borders of STN. To summarize, using the most advanced and most-recent technique, the challenge of designing a data-driven model for detecting both the entry and exit borders of STN in an intraoperative manner is an unmet need. The existing problems are: (a) limited data to be used for generation of the model; (b) the need for using offline techniques and normalized features that require critical post-operative manipulation; and (c) limited machine learning power due to the use of classical techniques for the generation of the data-driven physiological model that can represent the borders of STN. Therefore, in this paper, we propose to address the challenge through: (a) collection of a rich and unique data-set to be used for evaluating the possibility of reaching high accuracy intraoperatively; (b) using features that can be calculated intraoperatively with no need of critical postoperative normalization; and (c) relying on the power of the collected dataset and using a state-of-the-art strong machine learning algorithm (i.e. deep neural network) to model the nonlinear neurophysiology in order to model the borders of the STN. This paper, reports, for the first time, that using data-driven models, it is feasible to get an accuracy higher than 90% for localization of STN intraoperatively. In addition, some preliminary feasibility results on the performance of basic classic linear classifiers were presented in [18]. These results are in line with the conventional literature review given above.

In this paper, a new technique is proposed to automate the process of localization of STN and to find the dorsal and ventral borders of the nucleus during DBS surgery. For this purpose, we collected 713 microelectrode tracks from DBS surgeries of 100 PD patients. To the best of our knowledge, this is the largest data set collected for this purpose, which allows us to evaluate the possibility of using complex machine learning algorithms for modeling the neurophysiology based on which we can detect the borders of the STN. Currently, such an assessment is performed entirely visually by the clinician looking at the record and listening to the sound of the activity. Such an approach introduces significant subjectivity to the interpretation and can introduce error in localization. An autonomous STN localization tool that can provide objective feedback to neurosurgeons during the procedure would expedite the surgical procedure and improve placement consistency and accuracy.

The proposed learning technique utilizes a sizable database of clinical data collected and labeled in this study by expert neurosurgeons, which ensures its accuracy. In other words, the knowledge of placement locations and the corresponding MER recordings acquired during 100 surgeries is encapsulated in the training algorithm of the proposed technique. When implemented in the OR, the algorithm can reduce subjectivity of STN localization thereby directly having an impact on placement accuracy.

In this study, we evaluated the performance of several classical and modern classification methods for separating the signals that are from inside and outside the STN to detect its border based on electrophysiological activity. Three sets of different features were extracted from MER signals of 100 PD patients who had previously undergone DBS implantation. The first set of features corresponds to the conventional feature space which was used in [11], [13]. The second set of features denotes the Fast Fourier Transformation (FFT) features, and the third denotes wavelet transformation features. The main advantage of using FFT and wavelet features over conventional features is that they can be extracted intraoperatively since no postoperative step is needed. In [19], Snellings *et al.* mentioned that wavelet-drived background levels on STN were significantly higher than other regions and they can be a reliable source of information to identify the border of STN intraoperatively.

Deep neural networks and four classical machine learning algorithms (Support Vector Machine, Logistic Regression, K-Nearest Neighborhood and Decision Tree) were used for classification. In addition, to improve the accuracy a new design of an ensemble classifier consisting of four machine learning approaches was also applied to classify and predict the STN border.

The results of the comparative study, support the effectiveness of the designed technique in comparison to the existing methods in the literature. We show that the methods proposed in this paper not only significantly improve the accuracy of STN localization using MER signals but they can also be implemented intraoperatively to provide feedback for the surgical team. In this study, an accuracy of 92% was reported for STN border localization using wavelet transformation features and deep neural networks. As a result, the proposed technique has the potential to be used in the operating room for assisting neurosurgeons during DBS surgery to localize the STN.

Remark 1: In this paper, for the first time, it has been shown that by extracting wavelet transformation features and FFT features from microelectrode recording signals, it is possible to train a nonlinear data-powered computational deep neural network model to detect and decode the two borders of the STN. In this study, an accuracy of 92% was reported for STN border localization based on wavelet transformation features only. As a result, the trained model which is ready to use now, can be employed intraoperatively for assisting neurosurgeons during DBS surgery to localize the STN. Such intraoperative performance has not been achieved before. This work is the extension of the authors’ feasibility study presented in the conference format [18] in which a small dataset and partial features space were used to train preliminary linear models. Also, it should be noted that the numbers of extracted features in the FFT and wavelet transformation were 10,000 and 120,000 respectively.

## II. Methods and Materials

### A. Demographic Data and Data Acquisition

Microelectrode recordings were retrospectively acquired from 100 individuals with PD (38 female and 62 male), who had undergone DBS implantation with average age of $60\mp 6$ years. In total, 713 microelectrode tracks were used in this study as most patients received bilateral implantation of their DBS device. The retrospective review was approved by the local Human Subject Research Ethics Board (HSREB) office at the University of Western Ontario (REB # 109045). Prior to the surgery, all patients received T1 and T2 weighted MRI scans for surgical planning (Signa 1.5T, General Electric, Milwaukee, Wis). Target coordinates were calculated by first defining the anterior commissure and posterior commissure. The midline point was then used to plan the STN target; the initial stereotactic coordinates were: 12.0 mm lateral, 2.0 mm posterior and 4.0 mm ventral to the midline. Adjustments were then made according to the anatomy of the patient. All patients withheld their Parkinsonian medications for 12 hours before surgery.

**FIGURE 1.**
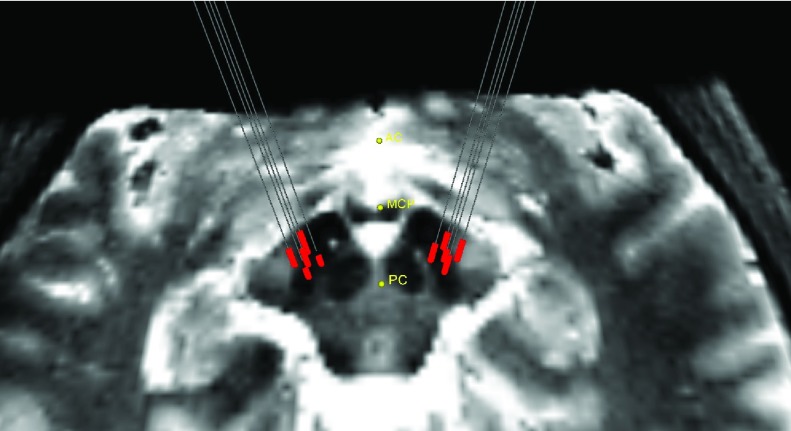
Microelectrode trajectory reconstruction. The red lines indicate the depths that the neurosurgeon decided the microelectrodes are inside the STN. The reconstructions and visualizations were performed using custom Python codes, the Visualization Toolkit, and 3D Slicer v4.8 (https://www.slicer.org). T2-weighted 7T images were co-registered to the preoperative CT image containing the Leksell frame. Images were converted to the NIFTI file format using dcm2niix [20]. Co-registration was performed using rigid registration tools in Niftyreg [21]. The coordinates of the microelectrode trajectories were extracted from Stealthstation (Medtronic Corp, MN).

**FIGURE 2.**
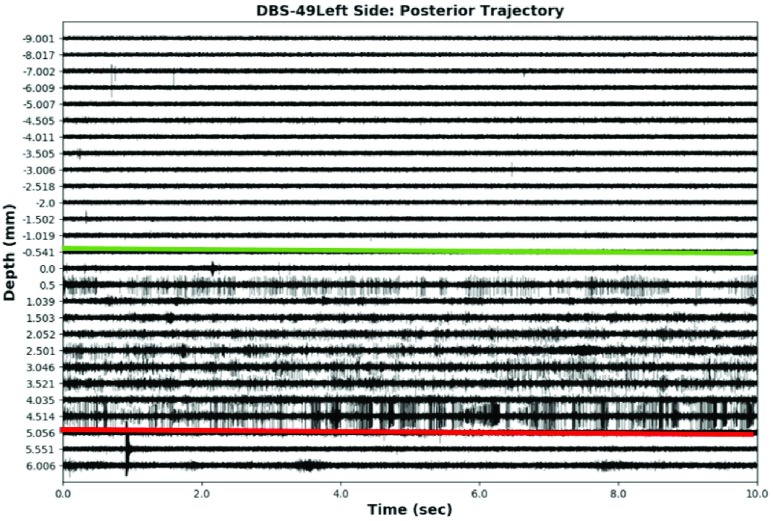
MER trace from an anterior microelectrode trajectory from an STN-DBS case at University Hospital, London Health Sciences Center. The microelectrodes were advanced from 10.0 mm to 5.0 mm in 1.0 mm intervals. From 5.0 mm to the end of the trajectory the unit was advanced in 0.5 mm increments. The green line indicates the dorsal border of the STN and the red line indicates the ventral border of the STN, as decided by the neurosurgeon.

On the day of surgery, the patients received a CT scan with the Leksell frame in place (Elekta Instruments, Sweden). Transferring the preoperative plan to the frame space was carried out by fusion of the stereotactic CT to the preoperative MRIs (StealthStation, Medtronic Corp, MN). The patients were then brought to the operating room, a sterile field was established, and a burr hole was drilled anterior to the coronal suture. A computer-controlled microelectrode drive was mounted to the Leksell frame (StarDrive, FHC Inc., Bowdoinham, ME), and 5 cannulas with stylets were lowered to 10.0 mm above the surgically planned target. The stylets were removed and replaced with 5 tungsten microelectrodes ($60~\mu \text{m}$ diameter) with an impedance of 0.5–$1.0~m\Omega $ at 1kHz (FHC Inc., Bowdoinham, ME). Microelectrode signals were then captured from 10.0 mm to 5.0 mm above the target in 1.0 mm steps. From 5.0 mm onwards, a step size of 0.5 mm was used. Once the ventral border of the STN was found the recordings were completed (generally around 4.0 mm to 5.0 mm below the surgical target. The neurosurgeon and electrophysiologist decided on the best microelectrode track, all microelectrodes were removed, and the final therapeutic electrode was introduced down the selected optimal trajectory. In Fig. 1 the trajectory of microelectrodes inside the brain is shown. At each recording site, data was collected for 10 seconds, which resulted in ~25–30 recordings for each microelectrode. The signals were sampled (24kHz, 8-bit), amplified (gain: 10000) and digitally filtered (bandpass: 500–5000 Hz, notch: 60 Hz) using the Leadpoint recording station (Leadpoint 5, Medtronic). All the computational analyses were conducted using custom scripts in Python and MATLAB. A sample MER signal from a right-side anterior trajectory is shown in Fig. 2. This figure demonstrates the difference in electrophysiological signal inside and outside the STN.

### B. Feature Extraction

Feature extraction plays an important role in biomedical signal processing. The features should provide meaningful information to the machine learning algorithms and be efficient in the computational step. In this paper, we implemented three different feature extraction methods: $(a)$ conventional post-operative features, $(b)$ fast fourier transformation, and $(c)$ wavelet transformation. A brief explanation of each method is as follows:

#### 1) Feature Extraction: Conventional Post-Operative Features

To compare the performance of the technique proposed in this paper with that of previous studies, we have extracted the most effective ten state-of-the-art features reported in [11], [13], and [12]. A list of these features for one 10-second interval is given below:

• Number of spikes per the 10-second intervals;
• Standard deviation of time differences between the spikes of the 10-second intervals;
• Pause index: the ratio the number of spikes greater than 50 ms to the number of spikes less than 50 ms;
• Pause ratio: the ratio of the total time of inter spike intervals greater than 50ms to the total time of those less than 50ms;
• Root Mean Square (RMS) value of the signal amplitude in the 10-second intervals;
• Spiking rate: number of spikes per unit time (one second).
• Teager Energy, which can be calculated as follows:
  \begin{equation*} E=\sum _{i=2}^{N-1}{x_{i}^{2}-x_{i-1}x_{i+1}};\tag{1}\end{equation*}
  where, $x_{i}\in X=\{x_{1},x_{2}.\ldots.x_{n}\}$ and N is the number of samples in each signal;
• Zero crossing: the number of zero crossings in each 10-second interval;
• Curve length: the sum of consecutive distances between points in the 10-second interval, as calculated below:
  \begin{equation*} L=\sum _{i=1}^{N-1}{|x_{i+1}-x_{i}|}\tag{2}\end{equation*}
• Threshold ($\gamma $):
  \begin{equation*} \gamma =\frac {3}{N-1}\sqrt {\sum _{i=1}^{N}(x_{i}-\bar {X}) }\tag{3}\end{equation*}
  where $\bar {X}$ is the average of the 10-second time interval.

**FIGURE 3.**
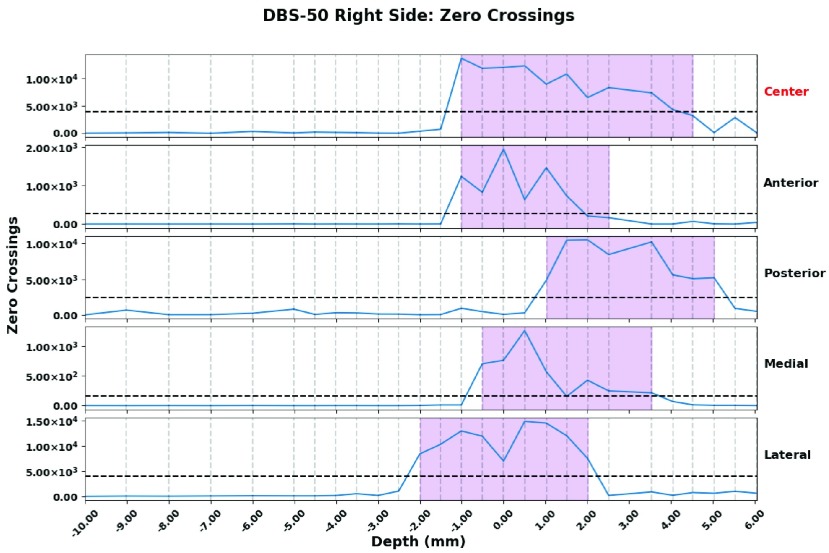
The purple shaded area indicates where the nucleus was determined to be based on the recordings. The red highlighted depth indicates which channel the surgeon decided to use. Each dotted line represents a recording depth. Negative depth values are above the nucleus, and positive values are below. The black horizontal dashed line indicates the mean of zero crossing value in each trajectory.

As reported in [11], [13], the above-mentioned features need to be normalized because of potential instability in feature calculation. In the normalization procedure, the mean is subtracted from the values of the calculated features and divided by the standard deviation of features in one trajectory. As a result, it is not possible to use these features intraoperatively. In order to apply the normalization step, recorded signals from the entire trajectory are required. This is not available while implanting the electrodes. Thus, these features can only be used as a postoperative validation method but they cannot help to localize the STN during surgery. One of the features, Zero Crossing, is shown in Fig. 3 and shown that the difference between the values from inside and outside of the STN nucleus is visible and clear.

#### 2) Feature Extraction: Fast Fourier Transformation

**FIGURE 4.**
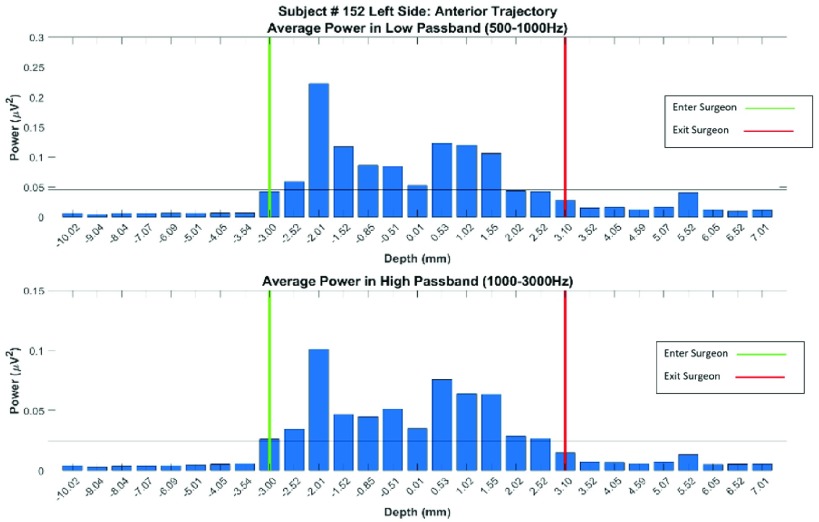
Power from DFT in two distinct frequency bands. The upper figure shows the frequency of 500–1000Hz indicating multi-unit activity, and the lower figure shows the frequency of 1000–3000Hz indicating single-unit activity. Negative depth values are above the nucleus, and positive values are below. The green line indicates the dorsal border of the STN and the red line indicates the ventral border of the STN, as decided by the neurosurgical team.

Fast Fourier Transformation is one of the methods which can provide valuable information in the frequency domain and also be computationally efficient. The frequency data provides information about where the power of the signals is concentrated. This is important since it is believed that the frequency content of neuron activities of each structure of the brain is distinctive. As a result, extracting FFT features from the MER signals can give us meaningful information about the location of the electrode inside the brain. As shown in Fig. 4, an increase in the power spectral density of FFT is visible when the electrode is inside the STN. It should be noted that by using FFT features, there is no need for the post-normalization step. FFT based features can be used intraoperatively. As a result, FFT-based feature space can be calculated while recording the MER signals during the operation while the neurosurgeon is implanting the electrodes. This is an advantage of the FFT-based feature space in comparison to the ones used in the literature (such as [11], [13]).

#### 3) Feature Extraction: Discrete Wavelet Transformation

The discrete Wavelet Transformation (DWT), similar to the Fourier transformation, gives the frequency content of the signal and also overcomes the drawback of losing time content in FFT. As a result, the extracted wavelet coefficients provide the energy distribution of the signal in time and frequency [22]. Furthermore, there are different types of wavelet mother functions which give us more options to extract features from signals. Mathematically, DWT is given by

\begin{equation*} W(u,2^{j})=\sum _{n=-\infty }^{\infty }s(n) \frac {1}{2^{j/2}}\psi \left({\frac {n-u}{2^{j}}}\right)\tag{4}\end{equation*}

where $\psi $ is the mother wavelet (basis function), $u$ represents time and $2^{j}$ is the scale parameter for the frequency axis [22]. The signal is down-sampled by 2 to the power level of ($2^{j}$). In our work, Haar wavelet mother functions were used with 4-level decomposition. Haar is a discrete mother function which is used for analysis of signals with sudden transitions, in this case spikes in the microelectrode signals.

## III. Classifiers

After the feature extraction step, the performance of an ensemble of multiple supervised classifiers and deep neural network was evaluated in locating the STN. The data was collected from 100 PD patients, and the outcome of the classifiers shows the label of the MER signals which is either zero or one. The signals labeled zero indicate that the microelectrode recordings are from outside of the STN nucleus and those labeled one are recorded from inside the STN. These outcomes were compared to the labels provided by the neurosurgeon (with experience of more than 200 DBS surgeries) for evaluating the accuracy.

### A. The Ensemble of Multiple Classifiers

As an alternative to the modern classifier, in this paper we have evaluated a specific ensemble of conventional classifiers consisting of

1) Support Vector Machine (SVM)
2) Logistic Regression (LR)
3) K-Nearest Neighborhood (KNN)
4) Decision Tree (DT)

Using the ensemble of these classifiers can help to enhance the accuracy and better deal with nonlinearity in the data. We also evaluated the performance of each classifier used in the ensemble technique separately.

There are different strategies for combining the classifiers. Among the existing techniques, “majority vote” is a commonly used approach. There are also other combination strategies such as “boosting” and “bagging” based on the majority vote method [23]. A weighted majority vote method is used for combining the classifiers here.

#### 1) Weighted Majority Vote Rule

In the majority vote scheme, the final decision goes with the one that has consensus for it or the one for which more than fifty percent of the individual techniques agree. If each of the classifiers does not give identical classification accuracy, then it is reasonable to attempt to give the more competent classifiers more weight in making the final decision. This method is called ”weighted majority vote rule” The formula for a weighted majority vote is:

\begin{equation*} y=arg \underset {i}{max}\sum _{j=1}^{m}w_{j}\chi _{A}(C_{j}(x)=i),\tag{5}\end{equation*}

where $\chi _{A}$ is the characteristic function $[C_{j}(x)=i \in A]$, and A is a set of unique class labels. $w_{j}$ is the weight assigned to classifier j based on its accuracy.

As a result, in this study, we evaluated the performance of an ensemble classifier approach which is based on the weighted majority vote rule and is composed of SVM, LR, KNN, and DT. All results, including the performance of the proposed ensemble technique in addition to the performance of each classifier used in the ensemble technique are given in Section IV.

### B. Deep Neural Networks

Artificial Neural Networks (ANNs) have become a popular classifier due to their inherent characteristics such as self-learning, robustness, adaptivity and generalization capability. ANNs are useful especially when there is enough data for training to obtain a good network. They denote a nonlinear mapping between inputs and outputs through multiple layers of neurons that are fully connected to each other. In the training phase, the ANN adjusts to get proper weights and bias to fit the database and produce the desired mapping between inputs and outputs. Processing information is as follows: each neuron in the input layer takes a sample from the dataset, multiplies it by a weight, adds a bias value and then passes it to the hidden layer. The hidden layer transforms this data by applying an activation function. Here is the mathematical representation:

\begin{equation*} h=f\left({\sum _{p=1}^{n}{W_{p}}^{2}f\left({\sum _{q=1}^{m}W^{1}_{pq}X_{q} + b_{p}^{1}}\right)+b^{2}}\right),\tag{6}\end{equation*}

where $W_{pq} (q=1,2,\ldots,m; p=1,2,\ldots,n)$ is the matrix of weights which expresses the weights between a neuron in the input layer and another in the hidden layer, $n$ is the total number of hidden neurons and $m$ is the number of input neurons. Also, $X$ is a vector of values in the input layer, $b$ is the bias and f is the activation function. A Deep Neural Network (DNN) is a multilayer neural network with several hidden layers capable of discovering unknown feature coherences of input signals. It works best with a large amount of data. In this study, due to the uniqueness and the size of collected data, we have collected over nine thousand microelectrode recordings as ten-second epochs from 100 patients, the DNN was used to model the nonlinear neurophysiology based on which the STN was localized. In order to find the most optimal DNN architecture for our problem, several different neural networks were trained and tested. To evaluate the performance of the multiple techniques in this work, we have divided the data set into two sections: 80% of the data was used for training based on ten-fold cross validation and 20% of the data was specifically used solely for measuring the performance. The architecture of the neural network that we chose in this study is a twelve-layer network with ten hidden layers and 50 nodes in each layer. When applying a deep neural network model, the concern of overfitting should be addressed. For this, here we employed two regularization methods: (a) weight decay term based on an L2 formulation, and (b) the dropout technique. L2 regression adds the square value of weights to the loss function in order to regularize the training process and avoid overfitting. The L2 norm is calculated as follows:

\begin{equation*} L_{2}=\lambda \sum _{i=1}w^{2}_{i}\end{equation*}

where $w_{i}$ is the weight of a hidden layer and $\lambda $ is the regularization term. A range of 0.001 to 0.1 was tested for $\lambda $ and the chosen final value which warranted the best performance was 0.0285. Dropout is also another regularization technique which disconnects some random nodes during the training. We have used a dropout rate of 0.3 which turns off 30% of the neurons from being trained during the process. All these parameters of the chosen DNN were chosen systematically by objectively tracking the accuracy of the network using ten-fold cross validation. All the computational analyses were conducted in Python 3.6 (TensorFlow library).

## IV. Results

**FIGURE 5.**
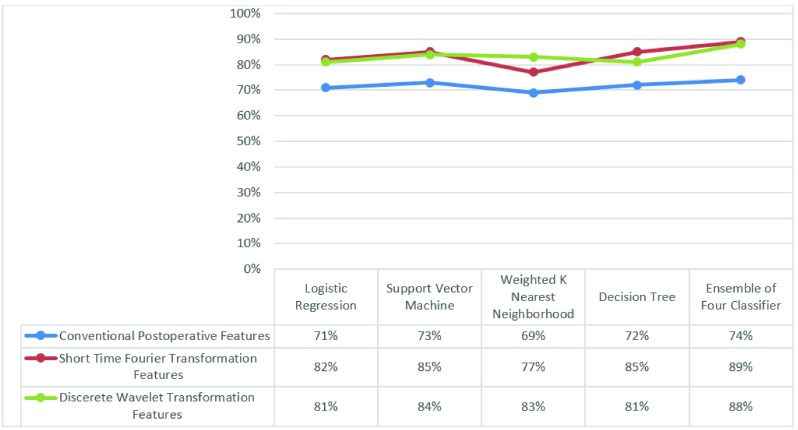
Accuracy of Classical Classifiers and Ensemble method for Localizing STN.

The dataset used in this study consisted of MER signals from 100 PD patients obtained during DBS surgery. Each recorded MER signal was a ten-second record from each trajectory and depth. On average, up to five microelectrodes were inserted on each side of a patient’s brain and in each, trajectory signals were recorded from 25 depths. As a result, the number of signals used in this study was large enough to support the use of a DNN architecture. All three feature sets (conventional postoperative features, FFT-base features, and wavelet features) were extracted from the signals. In the next step, the classifiers (SVM, LR, KNN, DT) were applied to separate the two classes (inside versus outside the STN). As mentioned, a DNN and a combination of classical classifiers were used in this study. To calculate and evaluate the performance of the proposed composite technique, the labels provided by the neurosurgeon during the operation were used. The results of this comprehensive comparative study are given in Figure 5. As can be seen in the figure, the ensemble classifier outperforms the single classical classifiers. For example, using FFT-based features, the accuracy of SVM alone is 85%, and this is the highest accuracy among the classical techniques, while using the proposed ensemble of all four classifiers, the accuracy can be improved to 90%.

The best results for this problem were achieved using the wavelet transformation and a DNN. This combination was able to separate the signals intraoperatively in terms of inside and outside the STN with an accuracy of 92%. It also had a low value of False-Positive (FP) rates equal to 3%. The confusion matrix for this trained DNN is shown in Table 1. All the trained algorithms were tested on new test MER signals and this showed that online real-time implementation allows input data to be processed within a short frame of time (less than 1.35 seconds), and provides feedback on location of electrodes at each depth with respect to the STN. As mentioned before, the major problem with conventional features is that they cannot be used during surgery because they need some postoperative normalization steps. As a result, although these features can provide postoperative validation, they cannot be used during the surgery to localize the STN. However, FFT-based features and wavelet features do not need a post-processing step and can be extracted in real-time during surgery. Thus, from the results shown in Table 2, the wavelet feature space extracted from the MER signals provides rich features for the DNN algorithm to assist the neurosurgeon in localizing the STN intraoperatively.

**TABLE 1:** Confusion Matrix for the DNN Model

		Actual placement	
		STN	Pre/Post-STN
Predicted placement	STN	True Positive Rate= 67%	False Positive Rate=3%
	Pre/post-STN	False Negative Rate=33%	True Negative Rate=97%

**TABLE 2:** Confusion Matrix for the DNN Model Based on Validation on the Unseen 20% Dataset

Features\Classifiers	Neural Network	Ensemble of Multiple Classifiers
Conventional Post-Operative Features	74%	76%
Short Time Fourier Transformation features	71%	67%
Wavelet Transformation features	85%	70%

In addition, it is important to note that regardless of the problem with intraoperative implementation of the conventional techniques, they do not have the accuracy of the ones proposed here. In this paper, two main approaches were proposed, (a) wavelet feature space used in a DNN, and (b) FFT-based feature space used in an ensemble SVM-LR-KNN-DT fused using a weighted majority vote. The accuracy of both approaches for localizing the STN was higher than conventional techniques. Unlike conventional techniques [11], [13], both approaches can be implemented intraoperatively with the wavelet-DNN having the highest accuracy.

Thus, from the results shown in Table 2, the wavelet feature space extracted from the MER signals provides rich features for a deep neural network algorithm for assisting neurosurgeons in localizing the STN intraoperatively.

Remark 2: It is worth highlighting that to evaluate the performance of the multiple techniques in this work, we have divided the data set into two sections: 80% of the data was used for training based on ten-fold cross-validation and 20% of the data was specifically used solely for measuring the performance. The main results reported in this paper are only for the 20% test set to evaluate the generalizability of the algorithm and strictly avoid the leak of information from the training set to the validation set. This result can be seen in Table 1. In addition, the results of the ten-fold cross-validation are given in Table 3.

**TABLE 3:** Results for the DNN Model Based on Ten-Fold Cross Validation on 80% Dataset

True Positive Rate= 73%	False Positive Rate=2%
False Negative Rate=27%	True Negative Rate=98%

## V. Conclusion

This study presented new techniques that can be used to assist a neurosurgeon during DBS surgery by providing accurate localization of the subthalamic nucleus (STN). For this purpose, Microelectrode Recording (MER) signals were processed and used in a machine learning algorithm. Based on this study, a combination of discrete wavelet transformation features and a deep neural network algorithm was suggested as a highly accurate approach to localize the STN during DBS surgery with an accuracy of 92%. To validate the performance of this approach, MER data from 100 patients living with Parkinson’s disease were used. A total of 9365 signals were recorded to construct the data set. A comparative study was conducted to evaluate the accuracy of the method in comparison with that of existing state-of-the-art techniques. The results showed that (a) the proposed approach can localize the STN with an accuracy of 92%; and (b) the technique described in this paper can be used as a cueing tool in the operating room to assist neurosurgeons to reach the STN target during DBS surgery in real-time.

It should be also noted that for the specific problem of STN localization, high true negative and low false positive values are very critical for the neurosurgical team In this regard the proposed DNN algorithm shows 95% precision. A future direction of this work will focus on further enhancing the performance by using automatic feature extraction and hybrid deep neural network algorithms. In this paper, we reported the performance of the proposed machine learning approach on the largest dataset that has been used in the literature to localize the STN based on MER so far. Collecting more data will allow for further investigations such as dividing the patients into test and validation groups instead of the data into test and validation sets, and more algorithmic development such as using a hybrid recurrent neural network (which may bypass the need for conducting feature extraction/selection phase will form a future line of research). Also, the trained STN localizer neural network will be released so that its performance can be tested by other groups on their datasets.
